# Cancer associated epigenetic transitions identified by genome-wide histone methylation binding profiles in human colorectal cancer samples and paired normal mucosa

**DOI:** 10.1186/1471-2407-11-450

**Published:** 2011-10-19

**Authors:** Stefan Enroth, Alvaro Rada-Iglesisas, Robin Andersson, Ola Wallerman, Alkwin Wanders, Lars Påhlman, Jan Komorowski, Claes Wadelius

**Affiliations:** 1 The Linnaeus Centre for Bioinformatics, Department of Cell and Molecular Biology, Science for Life Laboratory, Biomedical Center, Uppsala University, Box 598, SE-75124 Uppsala, Sweden; 2 Department of Immunology, Genetics and Pathology, Science for Life Laboratory, Rudbeck Laboratory, Uppsala University, SE-75185 Uppsala, Sweden; 3 Department of Surgical Sciences, Uppsala University, SE-75185 Uppsala, Sweden; 4 Interdisciplinary Centre for Mathematical and Computational Modelling, Warsaw University, PL-02-106 Warszawa, Poland; 5 Current address: Department of Immunology, Genetics and Pathology, Science for Life Laboratory, Rudbeck Laboratory, Uppsala University, SE-75185 Uppsala, Sweden; 6 Current address: Department of Chemical and Systems Biology, Stanford University School of Medicine, Stanford, California 94305, USA; 7 Current address: Department of Biology, Bioinformatics Centre, University of Copenhagen, Ole Maaloes Vej 5 DK-2200, Copenhagen N, Denmark

## Abstract

**Background:**

Despite their well-established functional roles, histone modifications have received less attention than DNA methylation in the cancer field. In order to evaluate their importance in colorectal cancer (CRC), we generated the first genome-wide histone modification profiles in paired normal colon mucosa and tumor samples.

**Methods:**

Chromatin immunoprecipitation and microarray hybridization (ChIP-chip) was used to identify promoters enriched for histone H3 trimethylated on lysine 4 (H3K4me3) and lysine 27 (H3K27me3) in paired normal colon mucosa and tumor samples from two CRC patients and for the CRC cell line HT29.

**Results:**

By comparing histone modification patterns in normal mucosa and tumors, we found that alterations predicted to have major functional consequences were quite rare. Furthermore, when normal or tumor tissue samples were compared to HT29, high similarities were observed for H3K4me3. However, the differences found for H3K27me3, which is important in determining cellular identity, indicates that cell lines do not represent optimal tissue models. Finally, using public expression data, we uncovered previously unknown changes in CRC expression patterns. Genes positive for H3K4me3 in normal and/or tumor samples, which are typically already active in normal mucosa, became hyperactivated in tumors, while genes with H3K27me3 in normal and/or tumor samples and which are expressed at low levels in normal mucosa, became hypersilenced in tumors.

**Conclusions:**

Genome wide histone modification profiles can be used to find epigenetic aberrations in genes associated with cancer. This strategy gives further insights into the epigenetic contribution to the oncogenic process and may identify new biomarkers.

## Background

Cancer has been traditionally considered a genetic and cytogenetic disease, but recent years have brought epigenetics to the forefront of cancer research [1,2]. Altered DNA methylation is nowadays considered a hallmark of neoplasia, including two different phenomena in cancer cells: global hypomethylation and CpG-promoter hypermethylation of tumor suppressor genes [1,2]. The importance of epigenetic alterations in cancer is further highlighted by their use in diagnosis and by the development of new therapeutic strategies aiming at correcting them [3].

Although histone modifications play major roles in processes such as transcription, replication and DNA repair, their oncogenic importance is not yet well established. However, several lines of evidence suggest that alterations in histone modifications are crucial in cancer development and progression. Global changes in histone H4 modifications seem to be universal markers of malignant transformation [4], while other histone marks predict the prognosis of prostate cancer [5]. Two of the most relevant histone modifications, both in general and from a cancer perspective, are histone H3 lysine 4 trimethylation (H3K4me3), which is found in promoters of active genes and histone H3 lysine 27 trimethylation (H3K27me3), which is preferentially associated with promoters of inactive genes [6]. SMYD3, a histone methyl transferase specific for H3K4, is over expressed in colorectal, hepatic and breast cancers, suggesting that H3K4 hypermethylation can occur at promoters of oncogenes [7]. MLL, another H3K4 methyltransferase, is frequently translocated in various forms of leukaemia [8] and the polycomb protein EZH2, a H3K27 specific histone methyl-transferase, is altered in multiple types of cancer [9]. Furthermore, recent reports suggest that H3K27me3 mediated silencing of tumor suppressor genes is a frequent event in prostate cancer [10,11].

Human genome-wide binding profiles for H3K4me3, H3K27me3 and other histone modifications have been previously generated, but in most cases, cancer cell lines were used as biological material [10,12]. Although this offers important insights into the functionality of the histone marks, it might not be optimal when investigating their importance in oncogenesis, as exemplified by the discrepancy between H3K27me3 profiles in normal tissue and cell lines of the same origin [13], or by increased DNA methylation in embryonic stem cells (ESCs) due to *in vitro *culture [14]. Therefore, in order to establish how chromatin is altered during the oncogenic process, histone modification profiles from normal and tumor tissue samples are most desirable. However, and to the best of our knowledge, this has only been reported in liver [15] and pheochromocytoma [16].

Colon adenocarcinomas display numerous epigenetic alterations, including hypermethylation of tumor suppressor genes and loss of imprinting at *IGF2/H19 *[17]. Interestingly, the major risk factor for CRC is age and epigenetic lesions accumulate with aging and contribute to cell transformation [18]. Despite the evident role of histone modifications in colon cancer, histone modification profiles have not been analyzed in coupled normal and tumor colon samples. Here we present the first genome-wide maps of H3K4me3 and H3K27me3 in paired normal and tumor samples from CRC patients. We identify previously unknown changes in the methylation statuses between the normal and tumor samples and correlate these to CRC-related pathway and function. Taken together, these data could be an important resource in understanding the epigenetic alterations associated to CRC.

## Results

### H3K4me3 and H3K27me3 binding profiles in paired normal and tumor samples from CRC patients

**Figure 1:**
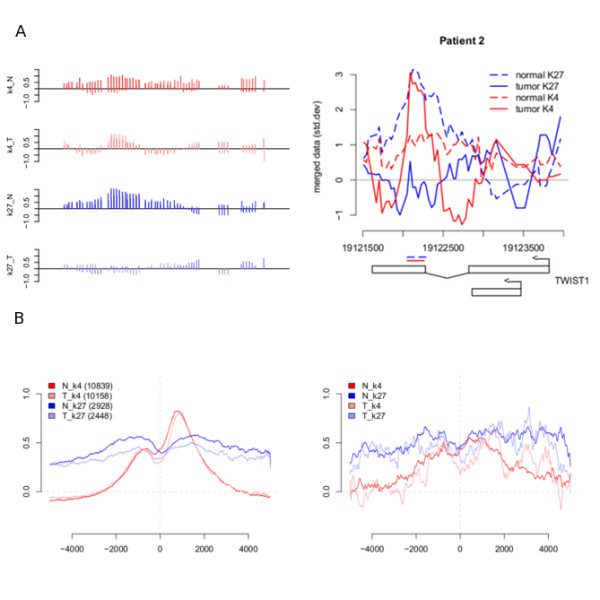
**ChIP-chip signals**. (A) Signal example from the *TWIST1-gene* (chr7) with data from patient 2. Left panel shows individual probes in replicates for all signals after analysis in TAS. Right panel shows normalized and averaged signal in terms of standard deviations (y-axis). Detected regions and location (HG18) of the *TWIST1* gene is also marked out. (B) Footprints of normal and tumor K4/K27 signal in patient 1 around the TSS's (left) of genes associated with enriched regions in each group separately and centred around genes associated with bivalent regions (right) in normal and tumor respectively.

We generated enrichment profiles for H3K4me3 and H3K27me3 in tumor samples from two CRC patients, for which the profiles in normal mucosa have previously been generated [13] and for the colon adenocarcinoma cell line HT29. After determining the enriched regions (Figure 1A) for each modification in normal and tumor samples (Table 1 and Additional file 1), they were annotated to human genes using the UCSC knownGenes databases [19] (Table 1) (Methods).

**Table 1:** Number of annotated geneSymbols and enriched regions.

		Regions	geneSymbols									
		
			Patient 1 Normal		Patient 1 Tumour		Patient 2 Normal		Patient 2 Tumour		HT29	
			K4	K27	K4	K27	K4	K27	K4	K27	K4	K27
Patient1 Normal	K4	6136	**6065**	431	4570	552	3898	450	3552	302	4323	387
	K27	3137	0	**1775**	191	926	190	1267	146	744	148	359

Patient1 Tumour	K4	6783	0	0	**7416**	344	4946	173	4430	119	5771	262
	K27	3177	0	0	0	**1981**	315	1078	244	756	315	457

Patient2 Normal	K4	5316	0	0	0	0	**5756**	174	3934	125	4535	248
	K27	3594	0	0	0	0	0	**1976**	119	877	143	422

Patient2 Tumour	K4	5670	0	0	0	0	0	0	**5406**	80	4106	251
	K27	3524	0	0	0	0	0	0	0	**1623**	112	430

HT29	K4	6544	0	0	0	0	0	0	0	0	**6938**	223
	K27	2612	0	0	0	0	0	0	0	0	0	**1494**

**Figure 2:**
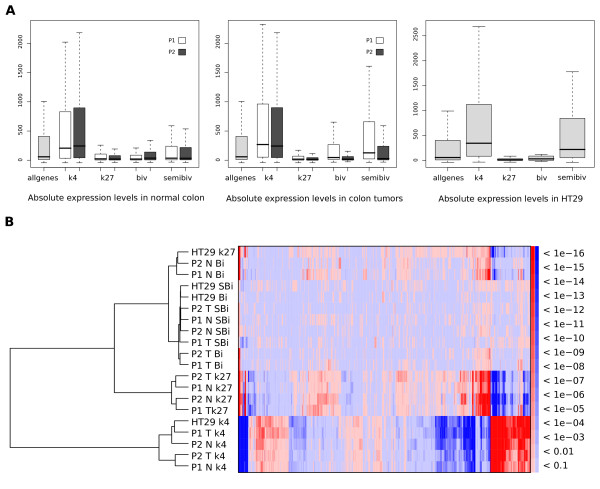
**Expression patterns and functional classification**. (A) Expression profiles in associated genes from normal patient material (left), tumor patient material (middle) and HT29 (right). In each group, the average expression on the entire array, the expression in K4, K27, bivalent and semi-bivalent genes respectively are depicted. (B) Hierarchical clustering of p-values of GO (Biological Process) terms. Over/under- representation is denoted by red/blue color and level of significance by color intensity. The three major clusters are made up by K4 fractions, K27 fractions and Bivalent/Semi-bivalent.

Signal footprints were created around transcription start sites and both the H3K4me3 and H3K27me3 patterns were similar to those reported [20-22] in other cell types (Figure 1B). As a quality control, microarray expression data from public repositories for normal colon, CRC tumors and HT29 were used [23]. Although the expression data comes from unrelated patient material, the objective was to study genome wide trends in transcription given the epigenetic status and not single gene effects where expression from the same patient material is essential. The expression of H3K4me3 enriched genes were higher than average, while H3K27me3 genes tended to be silent (Figure 2A). Bivalent and especially semi-bivalent (Methods) gene groups included fewer genes and were poorly expressed, although with higher variation in expression. The quality of our data is also supported by previous validation of H3K4me3 and H3K27me3 enriched regions by qPCR in normal colon [13] demonstrating that ChIP-chip analysis can be performed using human tissue as biological material.

For regions with associated transcripts, gene ontology (GO) annotations were obtained and an analysis was performed for over/under representation of terms compared to a whole genome background (Figure 2B, Methods). The H3K4me3 and H3K27me3 gene groups have opposite functions and bivalent and semi-bivalent gene groups lie closer in functional space to H3K27me3 than H3K4me3 genes, as also suggested by expression data. Therefore, bivalent and semi-bivalent genes were treated as part of H3K27me3 gene groups for most subsequent analysis. In general, H3K4me3 fractions displayed over-representation in metabolic processes while H3K27me3 fractions showed enrichment in developmental categories. The overrepresented categories in H3K4me3 and H3K27me3 genes were very similar in the HT29 cell line.

### H3K4me3 and H3K27me3 profiles are similar between normal and tumor samples but distinct compared to CRC cell lines

**Figure 3:**
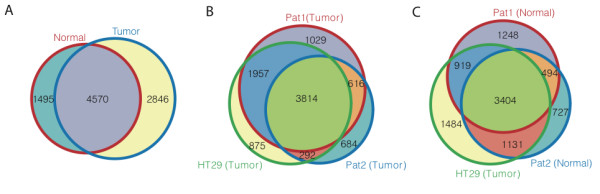
**Overlaps between genes marked with H3K4me3 in different samples**. (A) Overlaps between genes associated with K4 enriched regions detected in patient 1 normal and tumor tissue. (B) Overlaps between genes associated with K4 enriched regions detected in tumor tissue from patient 1, tumor patient 2 and HT29. (C) Same as (B) but for normal tissue from patient 1 and 2.

Using all probes we calculated the Pearson's correlation between all combinations of histone modifications, patient material (normal/tumor) and cell line (Additional file 2, Table S1). In all cases the correlation between any patient materials was always strongest when that material was compared to another patient material. In some cases, e.g. patient 1 tumor H3K27me3/K4me3, the correlation was highest with patient 2 normal H3K27me3/K4me3 rather than the HT29 cancer cell line. This pattern is more evident when regarding enriched regions only and by counting overlapping enriched regions, a relatively high similarity between normal and tumor samples and between patients for both H3K4me3 (Figure 3) and H3K27me3 was noted (Table 1 and Additional file 2, Figure S6). Around 60% of the H3K4me3 and 35% of H3K27me3 regions in both patients are common between normal and tumor tissue. When annotated transcripts rather than individual regions were used these numbers rise to around 70% and 50% respectively. Considering H3K4me3 genes, HT29 targets showed large overlaps with both patients normal and tumor samples (Table 1-2). The overlaps for the adenocarcinoma cell line SW480 [24] were quite poor (10-20%) both with tissue samples and HT29 (Table 2). We also noted that the number (around 1%, 80-431) of bivalent regions detected in promoters here is lower than the number detected in a previous study [25] on differentiated cells (CDC36+) where around 3% (693) of the genes were found to have bivalently marked promoters. Cui *et al *[25] performed their study using next generation sequencing and called more regions enriched throughout the genome suggesting that the main cause of difference lies in stringency of the downstream analysis.

**Table 2:** Comparisons between H3K27me3 target genes in colon tissue samples and CRC cell lines.

			Patient 1		Patient 2		Cell Lines	
			
			Normal	Tumor	Normal	Tumor	HT29	SW480
		
			K27	K27	K27	K27	K27	SUZ12
Patient 1	Normal	K27	**600**	310	399	237	138	74
	Tumor	K27		**660**	349	242	164	88

Patient 2	Normal	K27			**636**	282	154	84
	Tumor	K27				**512**	151	82

Cell Lines	HT29	K27					**512**	67
	SW480	SUZ12						**447**

The different gene categories considered (in bold) consisted of the genes marked by H3K27me3 in normal or tumor samples from patient 1 or patient 2, genes bound by H3K27me3 in HT29 CRC cell line and genes bound by SUZ12 in SW480 CRC cell line. Only genes were both H3K27me3 data in tissues samples and HT29 cell line and SUZ12 data in SW480 cell line were available were considered (total = 7393), which was limited by the coverage of the arrays used. The numbers in the diagonal of the table (in bold) indicate all genes included in each category, while the remaining numbers indicate the overlaps between each pair of categories considered in each case.

### Detection of genes and pathways with chromatin state differences between tumor and normal samples

**Figure 4:**
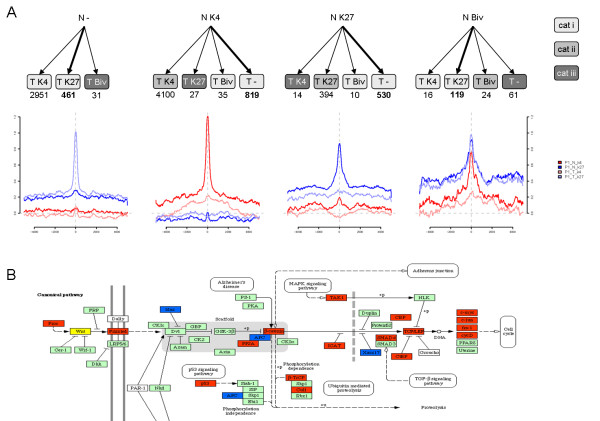
**Normal-to-tumor histone modification transitions**. (A) Normal-to-tumor transition graphs detected in patient 1 separated by starting condition. The number of associated genes is given below each of the 15 monitored transitions. The 15 transitions are grouped into 3 categories given by the color of the target. Below each start condition a region-centered footprint is drawn depicting the change in signal over the detected genomic regions. The selected transitions are highlighted in bold in the graph. (B) Part of the WNT-pathway color coded by change in epigenetic markers in patient 1. Red indicate an activating change ('N -' to 'T K4', 'N K27' to 'T K4' or 'N Biv' to 'T K4') while Blue indicate an repressive change ('N -' to 'T K27', 'N K4' to 'T K27', 'N K4' to 'T -', 'N K4' to 'T Biv' or 'N Biv' to 'T K27').

To identify the genomic regions differing in their chromatin state between normal and tumor tissues (Methods), the results were split in fifteen so called transitions each representing a combined state of one or more marks in normal and tumor. Three major groups were created; i) gaining or loosing a single mark, ii) keeping a mark and iii) changes involving both marks (Figure 4A and Additional file 2, Figure S4A). The majority fall into category ii), that is no change, and category i) which is in agreement with the large overlaps reported in the previous section (Additional file 2,Table S2).

Category iii) implies chromatin transitions between normal and tumor that can result in a switch from transcriptionally active (e.g. H3K4me3) to inactive states (e.g. H3K27me3) or vice verse. Therefore, it is the most interesting category in terms of potentially functional consequences from an oncogenic point of view. However, only 4 genes were common to both patients in category iii) (Additional file 2, Table S2). *KLF7* (N K27 to T K4), a transcription factor belonging to the KLF family that plays critical roles in differentiation, development, and maintenance of tissue homeostasis [26], but, to the best of our knowledge, without a specific role in colon cancer. The remaining three common genes (*EBF3*, *DKFZp667I0324* and *RBMS1*) were all bivalent in normal tissue but lost all their histone methylation in tumor. Among these genes, 2 have previously been functionally characterized. *EBF3 *is a transcription factor reported to be a tumor suppressor gene [27] with observed silencing in CRC cell line HCT116, was bivalent in normal and lost both marks in tumor. *RBMS1 *is a transcription factor that has been suggested as an important factor in inducing apoptosis in mice [28], and has been shown to have predictive power on colorectal cancer recurrence in human patient cohorts [29]. A number of transitions were specific to individual patients e.g. from category iii). Some genes had H3K4me3 in normal colon which changed to H3K27me3 in tumors, e.g. in patient 1 *HOXB13 *and *KREMEN2*, which are frequently down regulated in CRC [30,31]. Among genes with a silent state (H3K27me3 mark) in normal colon that became active in CRC samples (gained H3K4me3) there were several well-characterized oncogenes [32-36], e.g. in patient 1 *FLI1, WWTR1 *&*ZEB2 *and in patient 2 *TACSTD2 *&*TWIST1 *(Figure 1A and Additional file 2, Figure S5 (patient 1)), the last two with proposed importance in CRC [32,35].

Several genes in categories i) and ii) were found to have the same transitions in both patients. Both our patients have H3K4me3 on the *PTGER2 *gene in normal tissue and gained H3K27me3 in the tumor, which offers a mechanistic explanation to the previously reported expression pattern [37] of this gene in colorectal cancer. In addition, three genes common to both patients which carried bivalent marks in normal and lost H3K4me3 in tumor, *LHX9, PKNOX2 *and *LBXCOR1 *and one, *PRDM8*, lost the H3K27me3 in tumor, all of which are transcription factors with unknown function in colon.

The chromatin states of genes involved in biological pathways with relevance in CRC biology [38] was specifically investigated. The WNT-signalling pathway has been implicated in CRC [39,40], with the *APC *gene frequently silenced or mutated. Patient 1 has tumor repressive transitions in the *APC *gene and tumor activating transitions in *Axin, PP2A, β-catenin *and in many downstream factors along the WNT-signalling pathway affecting transcription, cell cycle and CRC development, especially within the *TCF*/*LEF *family [41,42] (Figure 4B). This suggests a repression on the β-catenin degradation complex and an up regulation of β-catenin dependent gene expression with downstream effects on the cell cycle [43]. Recently, a dual role of *APC *in the WNT-signalling pathway has been suggested with both activating and repressing mechanisms [44] which could explain the patterns found in patient 2 where *APC *has gained activating markers (Additional file 2, Figure S4B) In addition, patient 1 shows an activation of several known repressors of the WNT-pathway. *CtBP *represses E-cadherin which in turn is a tumor repressor restricting tumor cell motility and invasion [45]. The ubiquitin ligase *Cul1 *is part of a mechanism targeting proteins for degradation and thus an important factor in cell cycle control. Specifically, *Cul1 *acts as an oncogene that forms complexes that specifically target the cell cycle inhibitor *p27/Kip1 *for degradation [46].

### Global expression changes of H3K4me3 and H3K27me3 targets in CRC

**Figure 5:**
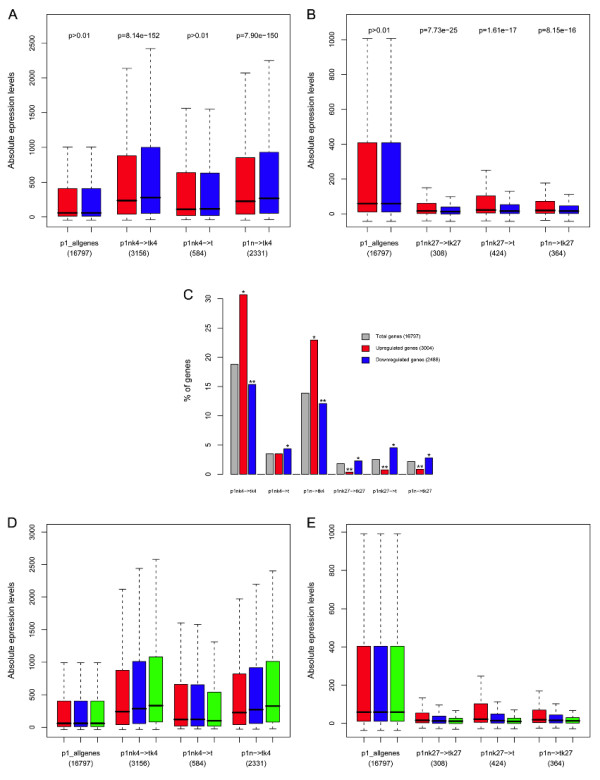
**Expression patterns in relation to histone modification transitions**. (A,B) Expression for paired normal colon (red) and tumor (blue) samples from 24 CRC patients was gathered from GEO public repository (GSE10950). Only genes where both expression and ChIP-chip data were available were considered. Boxplots indicate the absolute expression levels of all genes and those transition groups involving H3K4me3 (A) or H3K27me3 (B) for patient 1. Only transition groups with at least 100 genes were considered. The name of the transition groups and the number of genes in each group are indicated below the boxplots. For each group of genes, the expression of all genes in all 24 normal colon samples (red) was compared to the expression in all 24 tumor samples, with the p-value indicating the statistical significance of the expression being higher (A) or lower (B) in tumors than in normal samples, using a paired T-test. (C) Using the same expression dataset as in (A,B), genes that were significantly up regulated (red) or down regulated (blue) in tumors compared to normal samples were determined using a p-value < 0.001 (limma package, paired-sample design, multiple hypothesis corrected). Then, for each patient 1 transition group indicated in the X-axes, we calculated the percentage of genes in that group represented with respect to: total of genes (grey), up-regulated genes (red) and down-regulated genes (blue). Using a hypergeometric test we calculated if genes in the different transition groups were significantly over-represented (*) or under-represented (**) in the up/down-regulated genes compared to the distribution in all genes, using a p-value < 0.05 as cut-off. (D,E) For the same patient 1 gene groups as in (A,B), boxplots indicate the absolute expression levels in normal colon (red), tumor samples (blue) and HT29 cell line (green). Normal and tumor samples data is the same as in (A,B), while HT29 expression was obtained from GSM277543 dataset. The HT29 microarrays were analysed in parallel with the CRC samples, and all microarrays were normalized between each other.

Although epigenetic transitions within category iii) are expected to have important functional consequences, few examples are detected in a given CRC patient. Therefore, these examples are less likely to have large-scale changes in gene expression. More global relationships between chromatin state and gene expression were analyzed using all epigenetic transitions involving more than 100 genes i.e. maintenance of H3K4me3 or H3K27me3, tumor loss of H3K4me3 or H3K27me3 as well as tumor gain of H3K4me3 or H3K27me3. Microarray expression data for paired normal and tumor samples from 24 CRC patients [23] were used, and the expression changes between normal and cancer samples for the genes included in each transition category (patient specific) was compared (Figure 5 and Additional file 2, Figures S1-S3).

Genes in transitions where H3K4me3 was maintained or specifically gained in tumors, showed significantly higher expression levels in cancer samples (Figure 5A and Additional file 2, Figure S2). Opposite expression shifts were observed for genes that kept, gained or lost H3K27me3 in the tumor samples (Figure 5B, Additional file 2, Figure S2). The H3K4me3 and H3K27me3 gene groups also differed in the baseline expression in normal samples as H3K4me3 genes were highly expressed, while most H3K27me3 genes showed low expression levels. Furthermore, genes in H3K4me3 transition groups are typically already activated in normal tissue and become "hyperactivated" in tumors, while genes in H3K27me3 transition groups are lowly expressed in normal colon and become "hypersilenced" in cancer samples. The generality of the observations is supported by (i) the similar expression shifts obtained when considering the epigenetic transition groups from either patient 1 and patient 2 (Additional file 2, Figure S2A); and (ii) from a second expression data set with paired normal and tumor samples from 9 CRC obtained in a different array platform [47] (Additional file 2, Figure S2 B-C).

The significantly up- or down- regulated genes in cancer samples in the two expression microarray data sets described above assuming a paired sample design was analyzed further. For both patients and microarray data sets (Figure 5C and Additional file 2, Figure S3) genes keeping or gaining H3K4me3 in tumors were significantly over-represented among up-regulated genes and significantly under-represented among down-regulated genes. Opposite patterns were observed for genes that kept, gained or lost H3K27me3 in tumor samples (Figure 5C and, Additional file 2, Figure S3). These results indicate that at least for a fraction of genes the reported expression changes may be of large magnitude and occur in most patients. We gathered microarray expression data for HT29 [23] and considered the same epigenetic transitions as described above, and can report that the tendencies towards hyperactivation or hypersilencing of H3K4me3 and H3K27me3 gene groups became even more pronounced in HT29 cells (Figure 5D-E and Additional file 2, Figure S4).

### Hypersilencing of some H3K27me3 genes may involve loss of H3K27me3 and DNA hypermethylation

**Figure 6:**
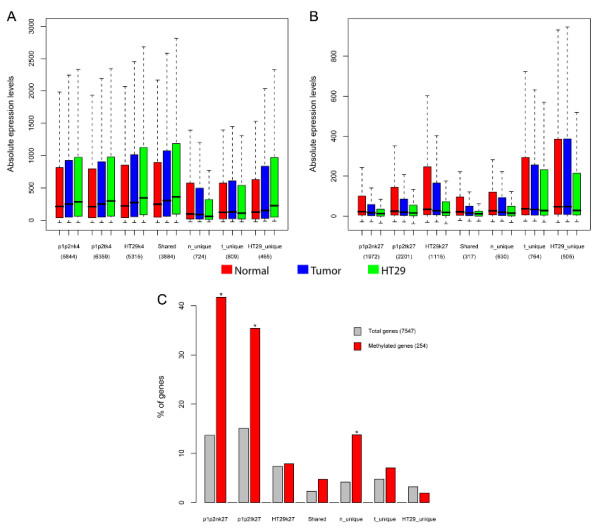
**Hypersilencing of some of the H3K27me3 target genes may involve loss of the histone mark and DNA hypermethylation**. In (A,B) the different gene groups presented in the bottom part of the figure correspond to all genes marked by H3K4me3 (A) or H3K27me3 (B) in both patients normal (p1p2n) or tumor samples (p1p2t) and in HT29 cell line. The Shared category included all those genes shared between normal, tumor and HT29 gene groups, either for H3K4me3 (A) or H3K27me3 (B). Similarly, the unique groups include those genes that are only found in normal (n_unique), tumor (t_unique) or HT29 (HT29_unique), when comparing the normal, tumor and HT29 gene groups. For each of these groups of genes, the expression values for normal colon (red), tumor samples (blue) and HT29 cell line (green) are indicated by boxplots and were obtained as described in Figure 4 D-E. In (C), only genes where both DNA methylation and ChIP-chip data were available were considered (Total genes = 7547), which was mainly limited by the coverage of the arrays used in the DNA methylation study. Then, for the same gene groups as in Figure 5B, we calculated the percentage of genes in each group represented with respect to the total of genes (grey) or those genes hypermethylated in CRC (red) as determined in Keshet *el al *[48]. Using a hypergeometric test we calculated if genes in the different gene groups were significantly over-represented (*, p.value < 0.01) among DNA hypermethylated genes compared to all genes.

The expression levels between normal colon, tumor samples and HT29 cell line for all H3K4me3 and H3K27me3 enriched genes were compared in the three samples types and for those genes that were shared or unique to any of them. The overall H3K4me3 or H3K27me3 gene groups showed progressive hyperactivation and hypersilencing in tumor and HT29 cells compared to normal colon, respectively (Figure 6A,B). This was also true for the genes with shared histone modification states, but less obvious in some of the unique groups. For the H3K27me3 unique groups, the hypersilencing pattern was still found for those genes carrying that histone mark only in normal colon samples (Figure 6B).

It has been recently reported [12] that genes enriched in H3K27me3 in normal prostate frequently become DNA hypermethylated and lose H3K27me3 in prostate cancer cell lines. Furthermore, DNA hypermethylation seems to be exacerbated by *in vitro *culture [14]. In order to test whether genes with the H3K27me3 mark in normal colon overlapped more with genes hypermethylated in CRC than genes that carried the histone mark in tumor samples, the lists of H3K27me3 genes was compared with those previously reported as frequently hypermethylated in CRC [48]. As shown in Figure 6C, both H3K27me3 genes in normal and tumor colon samples were significantly over-represented among DNA hypermethylated genes. This was not the case for the HT29 genes. Over-representation was only significant for normal colon, when considering H3K27me3 unique groups, and in fact even a trend towards under-representation was observed for H3K27me3 genes unique to HT29 (Figure 6C).

## Discussion

By comparing normal and tumor colon samples, it was observed that some regions, either shared or unique to one patient, showed transitions in the chromatin states that would suggest major functional consequences (i.e. from normal H3K4me3 to tumor H3K27me3 or vice verse). This resembles the results obtained after genetic mutation analysis of all coding sequences in CRC patients, where very few mutations are frequently shared between patients [49]. These observations suggest that in a given CRC patient, there could be a few major alterations (genetic and/or epigenetic) that can be crucial for tumorogenesis e.g. key components that could disturb the WNT-pathway such as silencing of the *APC *gene and activation of the β-catenin gene or up-regulation of repressors of tumor suppressing genes found in patient 1.

These few major and likely causative alterations could, however, have major impacts on global gene expression programs and chromatin states. Our observations suggest that H3K4me3 genes, typically active in normal colon, become "hyperactivated" in CRC. Since many of these genes are involved in basic cellular processes, their increased expression could merely be the result of higher metabolic demands and proliferation rates in tumor cells [50]. Mechanistically, a good candidate to mediate this "hyperactivation" response is *SMYD3*, a H3K4 methyltransferase frequently over-expressed in CRC and with oncogenic function [7]. On the other hand, H3K27me3 genes are already silent or lowly expressed in normal colon mucosa, and seemed to become "hypersilenced" in CRC. Down-regulation of H3K27me3 genes could be explained by polycomb-premarking of cancer-specific DNA hypermethylation [13,51] and subsequent loss of H3K27me3 and reduced epigenetic plasticity [12]. Due to tumor heterogeneity between different CRC patients, it is not expected that all normal colon H3K27me3 genes will loose the mark and become hypermethylated in a given tumor, which could explain why even genes that kept H3K27me3 state in our two patients seem to be hypersilenced when considering larger patient cohorts. Finally, silencing of genes showing specific gain of H3K27me3 in tumors has been also reported in prostate cancer [10,11]. Mechanistically, the silencing of all these H3K27me3 gene groups may involve *EZH2*, which is frequently over-expressed in CRC and other cancer types [9] and is able to interact with and recruit DNA methyltransferases [52].

Another important aspect of the work is the evaluation of how well cell lines may represent the chromatin states of normal or tumor tissues. According to our data, for H3K27me3, which is important in determining cellular identity [53], cell lines could give a misleading picture of chromatin states in tissues they are supposed to represent. Such discrepancy seemed to increase depending on the differentiation grade of the cell line, suggesting that low-grade cell lines could offer better cellular models than high grade poorly differentiated cell lines when studying chromatin states. Furthermore, our results and previous observations suggest that such alterations in H3K27me3 states can arise due to a combination of polycomb-premarking of DNA hypermethylation, subsequent lost of H3K27me3 and exacerbated DNA hypermethylation due to *in vitro *culture [12-14,51].

One such example is the polycomb binding profiles that were previously generated for the CRC cell line SW480 and used as a model of differentiated cells. As compared to ESCs, SW480 polycomb (H3K27me3) targets were not enriched in developmental related processes [24]. Here, and previously [13], we have shown that H3K27me3 marked genes, both in normal and tumor colon tissues, are largely enriched in developmental processes, and in fact, they significantly overlapped with ESC H3K27me3 targets. Therefore, we wanted to investigate how well CRC cell lines represent the chromatin states of colon tissue samples (Table 2). This might be expected since many H3K4me3 genes correspond to housekeeping genes typically active in any given cell type. On the other hand, when considering H3K27me3 gene groups, the overlaps between normal and tumor samples were quite high (40-60%), while somewhat lower for HT29 (20-30%), but as described above, H3K27me3 target genes in this cell line were still largely related to developmental processes. Moreover, the fact that the HT29 H3K27me3 profile, as compared to SW480, shows higher overlap with tissues and is enriched in developmental processes, can be explained by the fact that HT29 is a low grade CRC cell line (grade II), while SW480 is a high grade (grade III-IV), poorly differentiated and highly abnormal CRC cell line.

## Conclusion

In this work we generated the first coupled normal-tumor histone modification profiles in CRC, one of the cancer types with highest incidence in developed countries, that should provide a valuable resource for future chromatin studies with CRC and/or gastrointestinal focus. By studying histone modification profiles we show that it is possible to get new insights into the epigenetic process and discover new biomarkers for cancer. Two such candidates discovered here are the *PTGER2 *and *KLF7 *genes. The former gained H3K37me3 in tumor in both our patients and the latter changed its modification status from H3K4me3 to H3K27me3 in both patients thus changing from activated to repressed state. *PTGER2 *has previously been implicated in CRC but further research will be needed to clarify the role of *KLF7*.

## Methods

### Patient material

Normal colon mucosa [13] and tumor samples were obtained from two patients diagnosed with CRC at Uppsala University Hospital (Sweden). Immediately upon surgery, the removed material was kept on ice. Samples from the normal colon mucosa were taken at least 15 cm from the localized tumor, while the tumor sample was collected without containing parts of the normal mucosa. A gastrointestinal pathologist performed sample collection and tumor evaluation. The patients did not receive any anticancer treatment before surgery. All patients treated for CRC in Uppsala are registered in the Swedish Colorectal Cancer Registry. When admitted to the hospital they are asked to participate in that registry and also informed that biopsies will be saved in a biobank. After having read the information the informed consent is verbally obtained. Linked to this we have an ethical approval to perform studies like this, #2006/077.

### ChIP and ChIP-chip

ChIP on patient samples and HT29 cells were performed as previously described [13,54]. DNA amplification, fragmentation, labelling and hybridizations of ChIP and input DNAs were performed according to Affymetrix recommendations and basically as previously described [13], using Affymetrix GeneChip Human Promoter 1.0 arrays, which cover approximately from 7.5 kb upstream to 2.45 kb downstream of transcription start sites for over 25,500 human promoters. Raw array data has been deposited in ArrayExpress under accession number E-TABM-533

### Data analysis pipeline

Affymetrix Tiling Analysis SDK revision 4 was used to quantile normalize each pair of replicate ChIP measurements together with the two input measurements and adjusted to have a median intensity of 200 [55,56]. Replicates were combined using a sliding window of 301 bp (bandwidth = 150) assigning the median value of the replicates in the window to the center probe. From this point, all analysis was carried out using custom scripts in R [57]. Log2-ratios of merged ChIP measurements over merged input measurements were used for further analysis. Enriched regions were defined using a Z-score based sliding window approach. For each probe on the array, a Z-score was calculated from the average of log2-ratios of probes within a window of 150 base pairs centred on the probe against the distribution of log2-ratios on the array.

Enriched regions were defined from enriched probes (Z-score >= 6) using the following criteria; (i) at least 2 enriched probes within the region, (ii) each enriched probe must be flanked on both sides by existing measurements within 110 base pairs (iii) the maximum gap between enriched probes within a region is 110 base pairs. Regions were prolonged with probes positioned within 75 base pairs of the probes defining the tails of the regions. Finally, regions were merged if the shortest distance was less than 1000 base pairs.

Bivalent regions were defined as regions sharing at least three probes between H3K4me3 and H3K27me3 regions out of which at least two were enriched. Regions classified as having changed their methylation status ('transitions') between normal and tumor where required to have at least three probes in common out of which two needed to be commonly labelled as enriched in the different states. In addition to this we required a Z-score of at least 3 between the normal and tumor in the non-changed methylation status ensuring that we are not selecting regions immediately on opposite sides of the chosen cut-off, i.e. clearly separating changes involving bivalent marks to single marks from instances where a single mark is changed to another single mark.

### Gene Annotation

We associated enriched regions to UCSC knownGene transcripts if; (i) the regions were intragenic and positioned within 2.5 kb downstream of TSS or (ii) intergenic and positioned within 2.5 kb upstream of TSS but not associated to any other transcript through intragenic positioning. This window size (+/- 2.5 kb) was chosen based on the distribution of both H3K4me3 and H3K27me3 signals around transcription start sites. We defined transcripts to be marked by H3K4me3 if associated with at least one H3K4me3 region and no H3K27me3 region. The same was done for H3K27me3 associated transcripts. Transcripts associated with both H3K4me3 regions and H3K27me3 regions but not with bivalent regions were called semi-bivalent.

### Gene Ontology (GO) analysis

GO terms were tested using a one-sided Fishers exact test. P-values were corrected for multiple hypotheses testing using Bonferroni correction. The hierarchical clustering was done using Euclidean distances and the Ward agglomeration method. All analysis was done using R [57]

### Microarray Gene expression data sets

Microarray genes expression data sets were downloaded from Gene Expression Omnibus (GEO) public repositories. Expression data was obtained and analyzed using GEOquery and the 'limma' Bioconductor package in R [57]. Expression data from paired tumor and normal colon mucosa samples from 24 and 9 different CRC patients corresponded to GSE10950 and GSE5364 datasets, respectively. HT29 expression data (GSM277543) was originally generated in parallel with the tissue samples from GSE10950 dataset.

## List of abbreviations

CRC: colorectal cancer; H3K4me3: histone H3 lysine 4 trimethylation; H3K27me3: histone H3 lysine 27 trimethylation; ChIP: chromatin immunoprecipitation; ChIP-chip: chromatin immunoprecipitation and microarray hybridization.

## Authors' contributions

SE and ARI analysed the data, SE and RA designed and implemented the bioinformatics pipeline, ARI and OW prepared and performed the ChIP, AW and LP prepared the patient material, JK supervised the bioinformatics development. CW conceived and designed the study. All authors read and approved the final manuscript.

## Pre-publication history

The pre-publication history for this paper can be accessed here:

http://www.biomedcentral.com/1471-2407/11/450/prepub

## Supplementary Material

Additional file 1 **Marked genes: Lists of genes enriched with H3K4me3 or H3K27me3 in patient material or cell line**. Click here for file

Additional file 2 **Supplementary Material: 6 figures and 2 tables**. Click here for file
