# Supplementary material for: Cancer associated epigenetic transitions identified by genome-wide histone methylation binding profiles in human colorectal cancer samples and paired normal mucosa
Source: BMC Cancer. 2011 Oct 19;11:450. doi: 10.1186/1471-2407-11-450 (PMC3216894; doi:10.1186/1471-2407-11-450)
Supplement: Additional file 2 — Supplementary Material: 6 figures and 2 tables. [file 1471-2407-11-450-S2.DOC]

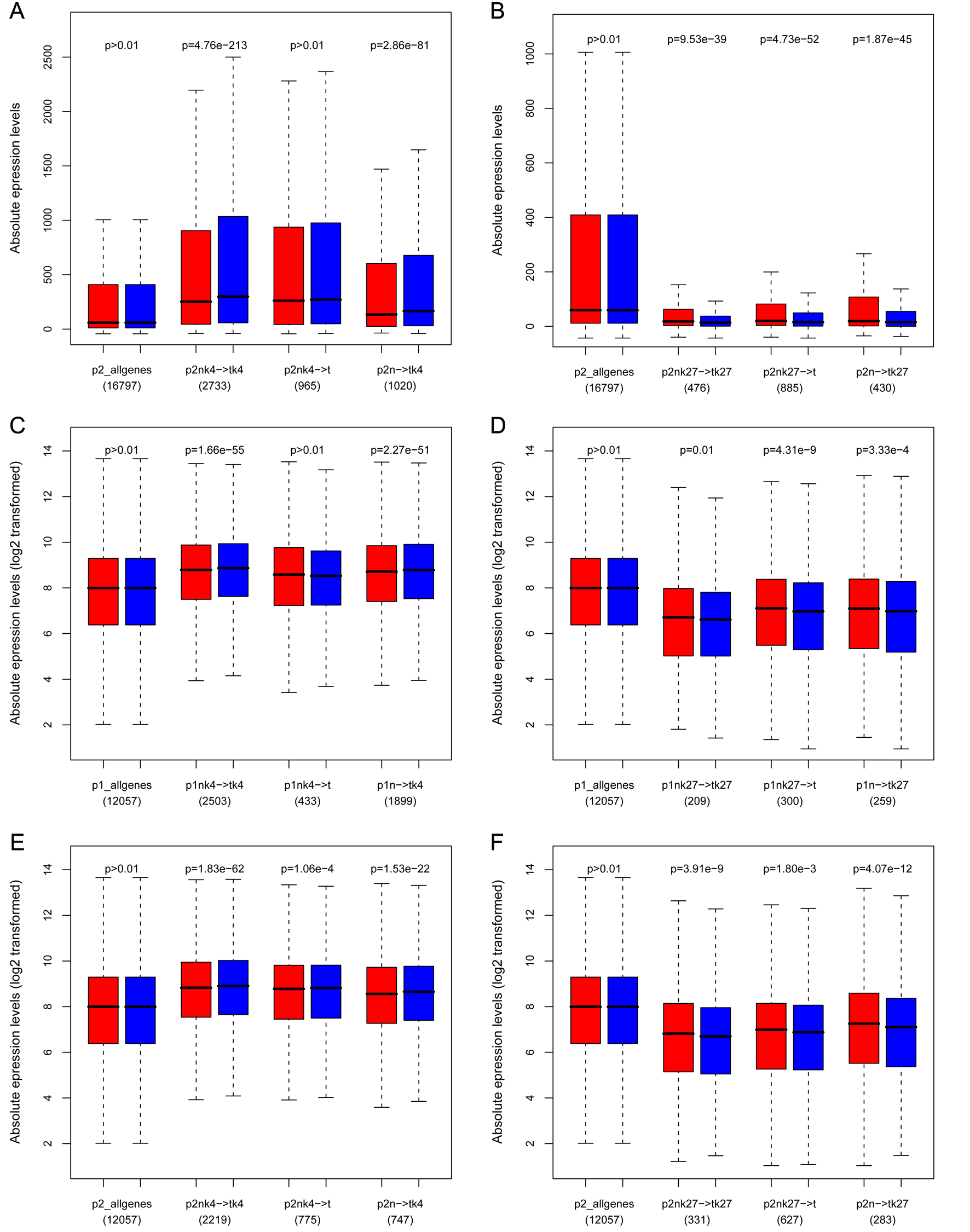


Supplementary Figure 1. Validation of the hyperactivation and hypersilenicng patterns of H3K4me3 and H3K27me3 targets genes, respectively, in CRC.

All panels in this figure were basically created as indicated in Figure 4 A-B. In (A,B), the expression of all genes or the indicated transition groups from patient 2 is represented by boxplots. Expression values were obtained from paired normal colon (red) or tumor samples from 24 CRC patients (GSE10950). In (C-F), a different microarray expression dataset (GSE5364) was used, consisting of paired normal colon (red) and tumor samples (blue) for 9 CRC patients. Transition groups for either patient 1 (C,D) or patient 2 (E,F) were considered with this second expression data set. For each gene group, p-values indicate the statistical significance of the expression being higher (A,C,E) or lower (B,D,F) in tumors than in normal samples, using a paired T-test.


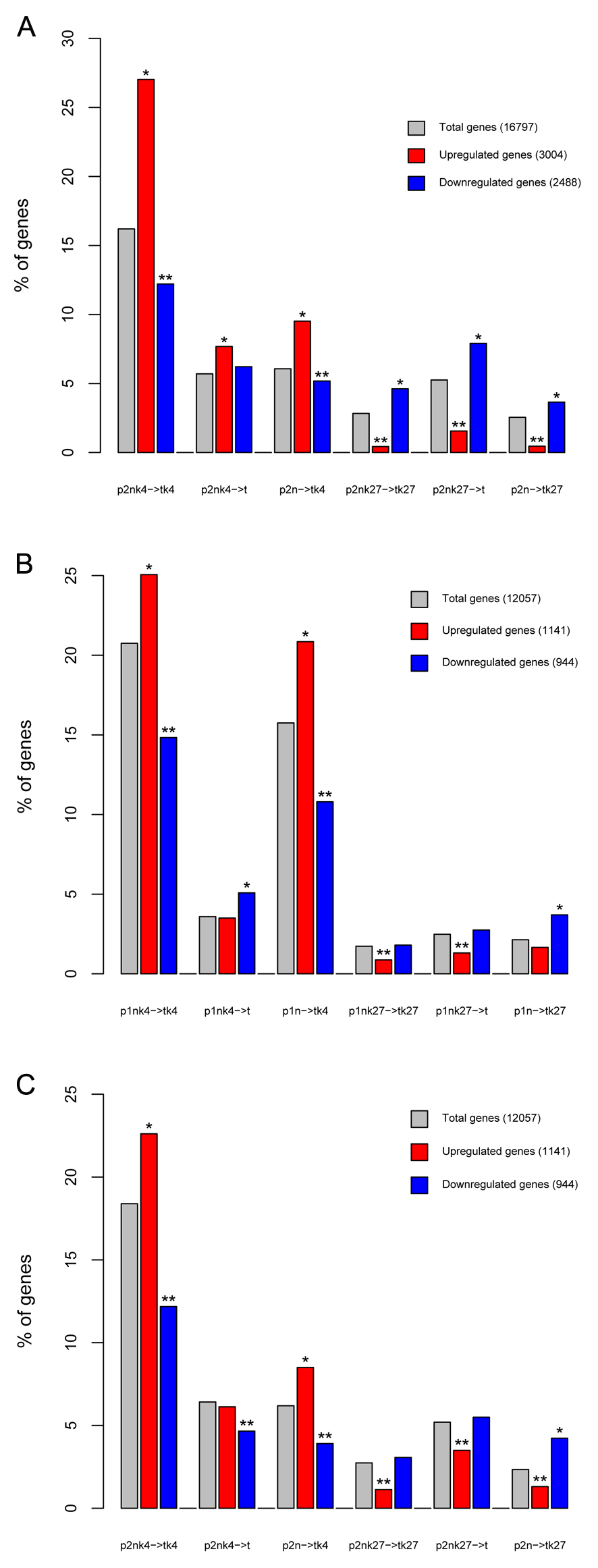


Supplementary Figure 2. Significant over-representation and under-representation of H3K4me3 and H3K27me3 genes, respectively, among CRC up-regulated and down-regulated genes. Using the expression data from paired normal colon and tumor samples from 24 CRC patients (A, GSE10950) or 9 different CRC patients (B,C, GSE5364), genes significantly up or down regulated in tumor samples compared to normal colon were determined. For GSE10950, a multiple hypothesis corrected p-value <0.001 was considered, while for GSE5364 p-value<0.05 was used as cut-off (see material and methods). For each transition group indicated in the X-axes either from patient 2 (A,C) or patient 1 (B), we calculated the percentage of genes in each group represented with respect to the total of genes (grey), the up regulated genes (red) and down regulated genes (blue). Using a hypergeometric test we calculated if genes in the different transition groups were significantly over represented (*) or underrepresented (**) in the up regulated or down regulated genes compared to the total, using a p-value<0.05 as cut-off.


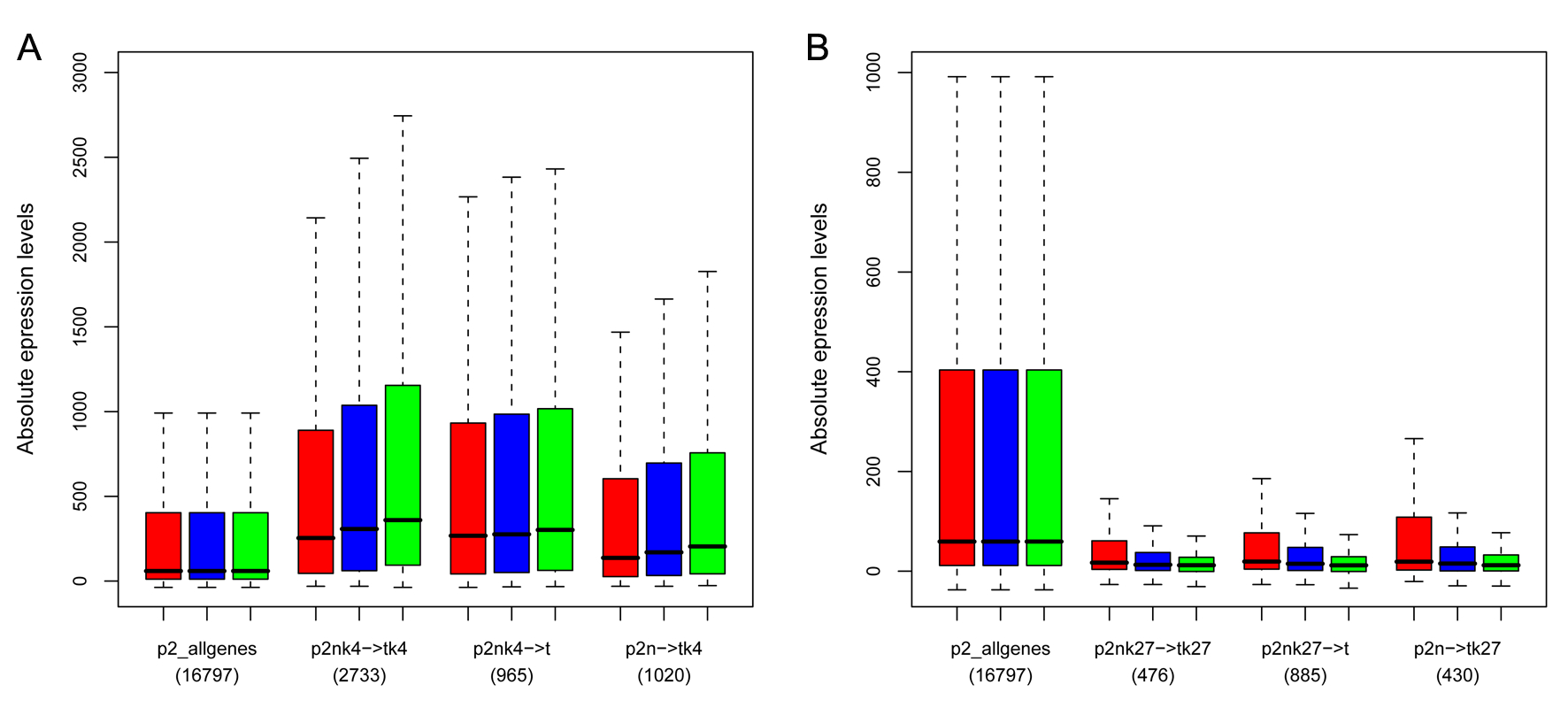


Supplementary Figure 3. Hyperactivation and hypersilencing patterns for H3K4me3 and H3K27me3 genes, respectively, in CRC are exacerbated in HT29 cell line.

The figure was created exactly as described in Figure 4 D-E, but transition groups from patient 2 were considered in this case.


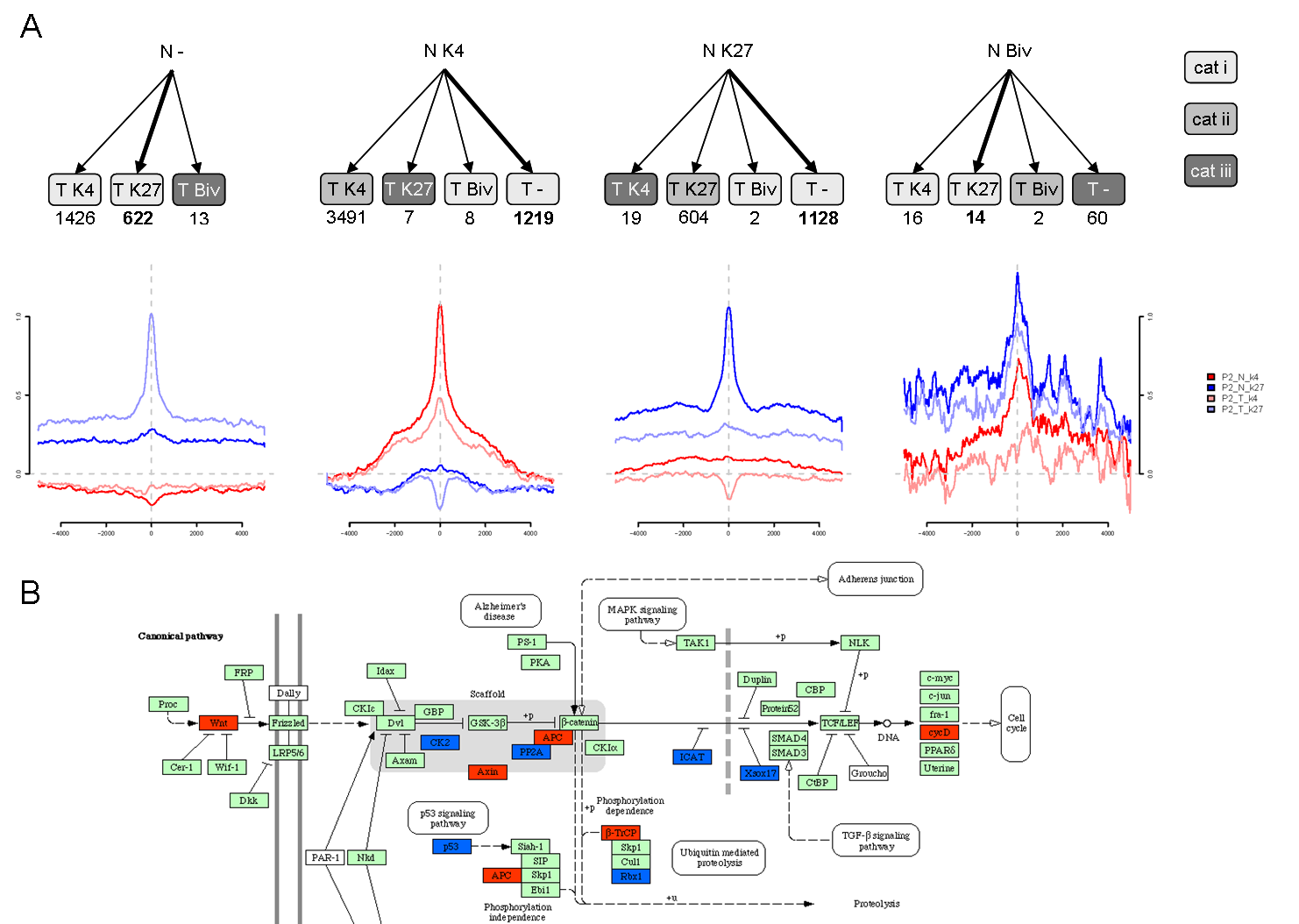


Supplementary Figure 4. (A) Normal-to-tumor transition graphs in patient 2 divided by the starting condition. The number of associated genes is given below each of the 15 monitored transitions. The 15 transitions are grouped into 3 categories given by the color of the target. Below each start condition a region centred footprint is draw depicting the change in signal over the detected genomic regions. The selected transitions are highlighted in bold in the graph. (B) Part of the WNT-pathway color coded by change in epigenetic markers in patient 2. Red indicates an activating change (‘N -‘ to ‘T K4’, ‘N K27’ to ‘T K4’ or ‘N Biv’ to ‘T K4’) while Blue indicate an repressive change (‘N -’ to ‘T K27’, ‘N K4’ to ‘T K27’, ‘N K4’ to ‘T -’, ‘N K4’ to ‘T Biv’ or ‘N Biv’ to ‘T K27’).


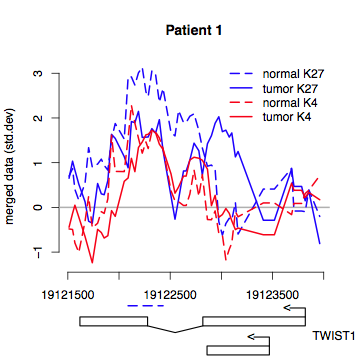


Supplementary Figure 5. Merged signal for the histone modifications over the two annotated TWIST1 gene isoforms for patient 1. Enriched regions called are drawn below the x-axis using the same color/line style as for the signals.


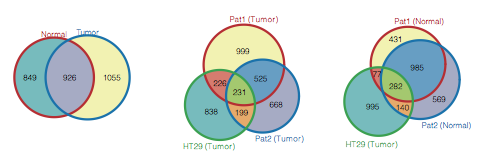


Supplementary Figure 6. (left) Overlaps between genes associated with K27 enriched regions detected in patient 1 normal and tumor tissue. (middle) Overlaps between genes associated with K4 enriched regions detected in tumor tissue from patient 1, tumor patient 2 and HT29. (right) Same as (B) but for normal tissue from patient 1 and 2.

|  |  |  | HT29 | | Patient 1 | | | | Patient 2 | | | |
| --- | --- | --- | --- | --- | --- | --- | --- | --- | --- | --- | --- | --- |
|  |  |  | Tumor | | Normal | | Tumor | | Normal | | Tumor | |
|  |  |  | K27 | K4 | K27 | K4 | K27 | K4 | K27 | K4 | K27 | K4 |
| HT29 | Tumor | K27 |  | 0.04 | 0.10 | 0.0 | 0.13 | 0.0 | **0.18** | 0.01 | 0.14 | 0.0 |
|  |  | K4 | 0.04 |  | 0.0 | 0.21 | 0.0 | **0.44** | 0.0 | 0.39 | 0.01 | 0.21 |
| Patient1 | Normal | K27 | 0.10 | 0.0 |  | 0.07 | 0.13 | 0.0 | **0.26** | 0.01 | 0.15 | 0.0 |
|  |  | K4 | 0.0 | 0.21 | 0.07 |  | 0.02 | 0.24 | 0.02 | **0.27** | 0.0 | 0.15 |
|  | Tumor | K27 | 0.13 | 0.0 | 0.13 | 0.02 |  | 0.08 | **0.25** | 0.02 | 0.15 | 0.0 |
|  |  | K4 | 0.0 | 0.44 | 0.0 | 0.24 | 0.08 |  | 0.01 | **0.46** | 0.0 | 0.21 |
| Patient2 | Normal | K27 | 0.18 | 0.0 | **0.26** | 0.02 | 0.25 | 0.01 |  | 0.07 | 0.23 | 0.02 |
|  |  | K4 | 0.01 | 0.39 | 0.01 | 0.27 | 0.02 | **0.46** | 0.07 |  | 0.0 | 0.26 |
|  | Tumor | K27 | 0.14 | 0.01 | 0.15 | 0.0 | 0.15 | 0.0 | **0.23** | 0.0 |  | 0.0 |
|  |  | K4 | 0.0 | 0.21 | 0.0 | 0.15 | 0.0 | 0.21 | 0.02 | **0.26** |  |  |

Supplementary Table 1. Pearson’s R^2^ between pairs of measurements, all 4.2 million probes where used in the calculation. Although essentially upper-triangular, the lower part of the table has been filled in to facilitate comparison between any pair of measurements. For each row, the strongest correlation between that row and column is indicated in **bold**.

| Category | Transition | Patient 1 | Patient 2 | Common | Genes |
| --- | --- | --- | --- | --- | --- |
| i | N- to T k4 | 2951 | 1426 | 451 |  |
| i | N k27 to T- | 530 | 1128 | 239 |  |
| i | N k4 to T- | 819 | 1219 | 71 |  |
| i | N- to T k27 | 461 | 622 | 63 |  |
| i | N Biv to T k27 | 119 | 14 | 4 | BC084573, LHX9, PKNOX2, LBXCOR1 |
| i | N k4 to T Biv | 35 | 8 | 1 | PTGER2 |
| i | N Biv to T k4 | 16 | 16 | 1 | PRDM8 |
| i | N k27 to T Biv | 10 | 2 | 0 |  |
| ii | N k4 to T k4 | 4100 | 3491 | 2469 |  |
| ii | N k27 to T k27 | 394 | 604 | 200 |  |
| ii | N Biv to T Biv | 24 | 2 | 0 |  |
| iii | N Biv to T- | 61 | 50 | 3 | EBF3, DKFZp667I0324, RBMS1 |
| iii | N k27 to T k4 | 14 | 19 | 1 | KLF7 |
| iii | N k4 to T k27 | 27 | 7 | 0 |  |
| iii | N- to T Biv | 31 | 13 | 0 |  |

Supplementary Table 2. Number of annotated transitions (geneSymbols) detected in either patient and common between the patients. For common transitions with at most 4 genes, the corresponding gene symbols are written out in the column “Genes”.
